# Supplementary material for: Metavinculin modulates force transduction in cell adhesion sites
Source: Nat Commun. 2020 Dec 17;11:6403. doi: 10.1038/s41467-020-20125-z (PMC7747745; doi:10.1038/s41467-020-20125-z)
Supplement: Supplementary file 1 — Supplementary Information [file 41467_2020_20125_MOESM1_ESM.pdf]

Metavinculin modulates force transduction in cell adhesion sites

Verena Kanoldt[1,2], Carleen Kluger[2], Christiane Barz[2], Anna-Lena Schweizer[1,2], Deepak Ramanujam[3,4], Lukas Windgasse[1], Stefan Engelhardt[3,4], Anna Chrostek-Grashoff[1,2], and Carsten Grashoff[1,2,*]

[1]Department of Quantitative Cell Biology, Institute of Molecular Cell Biology, University of Münster, Münster D-48149, Germany

[2]Max-Planck-Institute of Biochemistry, Group of Molecular Mechanotransduction, Martinsried D-82152, Germany

[3]Institute of Pharmacology and Toxicology, Technical University of Munich, 80802 Munich, Germany

[4]DZHK (German Centre for Cardiovascular Research), partner site Munich Heart Alliance, 80802 Munich, Germany

[*]Correspondence to: grashoff@wwu.de

Supplementary Information

Supplementary Figure 1-14

Supplementary Table 1-2

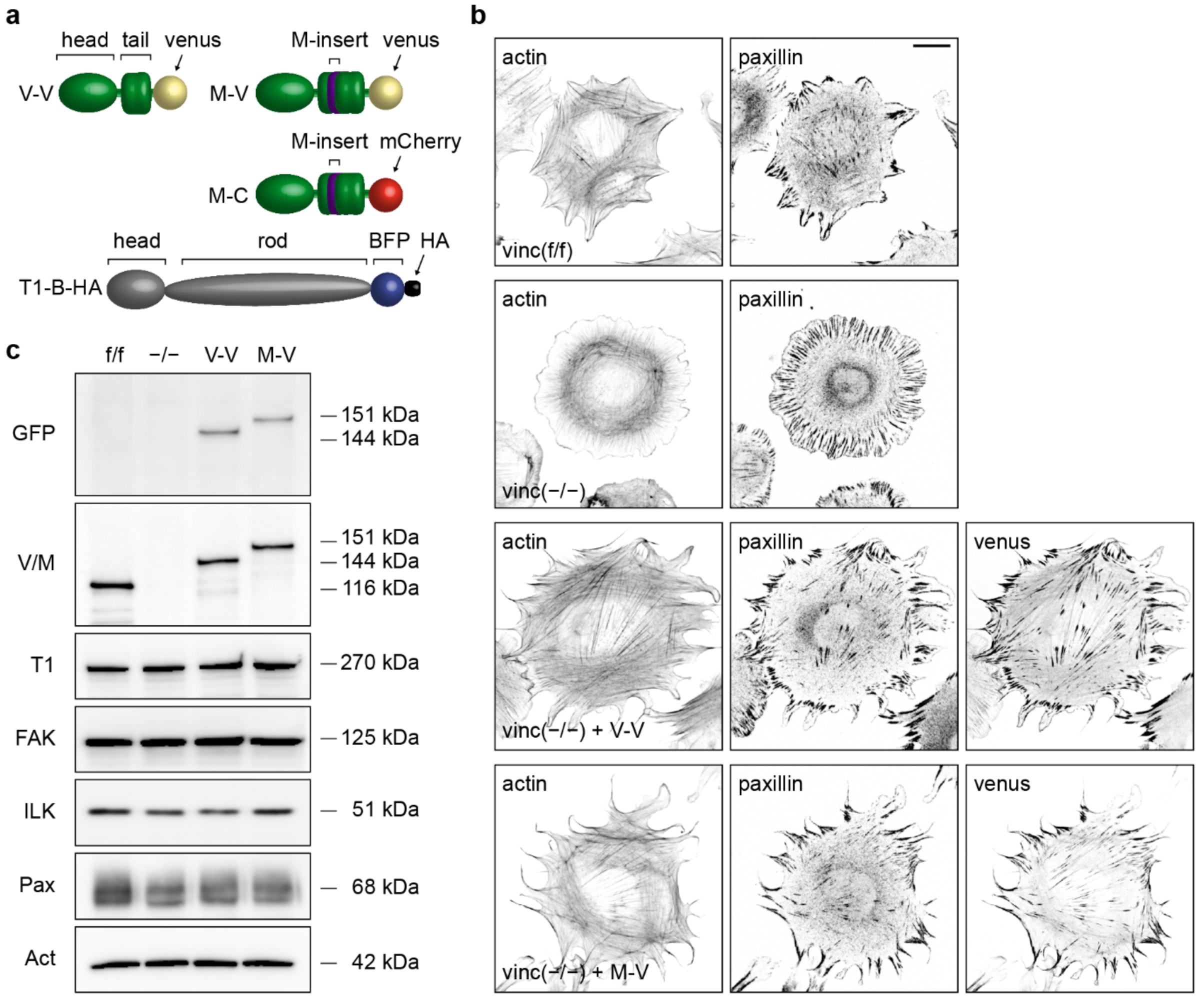

Supplementary Figure 1 Expression of vinculin and metavinculin in vinculin-deficient fibroblasts. (**a**) Schematic depiction of constructs used for localization, FRAP, and IP studies: vinculin (green) C-terminally–tagged with venus (yellow) (V-V); metavinculin C-terminally–tagged with venus (M-V) or mCherry (red) (M-C), and talin-1 (grey) C-terminally–tagged with TagBFP (blue) followed by an HA-tag (black) (T1-B-HA). Vinculin consists of a head and tail domain connected by a short flexible linker region. Metavinculin differs from vinculin by a 68-aa-long insert (purple) in the tail region. (**b**) Representative images of parental (vinc$^{(f/f)}$), vinculin-deficient (vinc$^{(-/-)}$), and vinc$^{(-/-)}$ cells reconstituted with V-V or M-V illustrate the rescue of cell morphology by the fusion proteins and their localization to FAs, which were visualized with a paxillin antibody in cells spreading for 4 h on FN-coated glass slides. F-actin was labeled with phalloidin. Images of cells reconstituted with V-V and M-V show individual channels of merge panels in Fig. 1a. Scale bar: 20 µm. (**c**) Western blot analysis of vinc$^{(f/f)}$, vinc$^{(-/-)}$, and vinc$^{(-/-)}$ cells reconstituted with V-V or M-V show that the tagged proteins are expressed to a similar extent, which is comparable to the endogenous vinculin level of the parental cell line. Expression levels of prominent focal adhesion proteins such as talin-1 (T1), focal adhesion kinase (FAK), integrin-linked kinase (ILK), and paxillin (Pax) are also similar. Sample processing controls

(Act) show that equal protein concentrations were used. Note that samples were derived from the same experiments and gels/blots were processes in parallel. Expected molecular weight values are indicated (kDa). Uncropped immunoblots with protein markers are provided in the Source Data file.

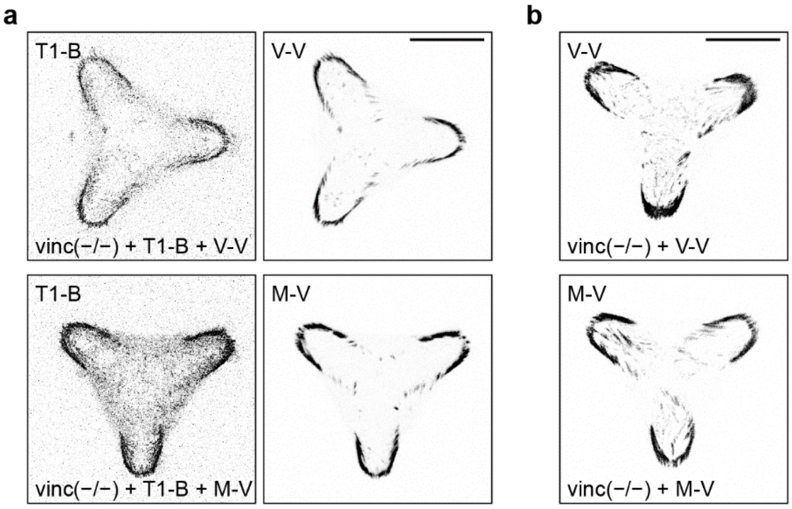

Supplementary Figure 2 Cells seeded on Y-shaped patterns for FRAP analysis. (**a**) Fluorescent images of vinculin-deficient (vinc$^{(-/-)}$) cells expressing talin-1-TagBFP-HA (T1-B-HA) and vinculin- or metavinculin-venus (V-V/M-V) seeded on a Y-shaped micropattern for FRAP analysis. (**b**) Fluorescent images of vinc$^{(-/-)}$ cells expressing V-V and M-V seeded on a micropattern. Cells form stationary and morphologically highly similar focal adhesions (FAs), which facilitates the FA-FRAP analysis. Scale bars: 20 μm.

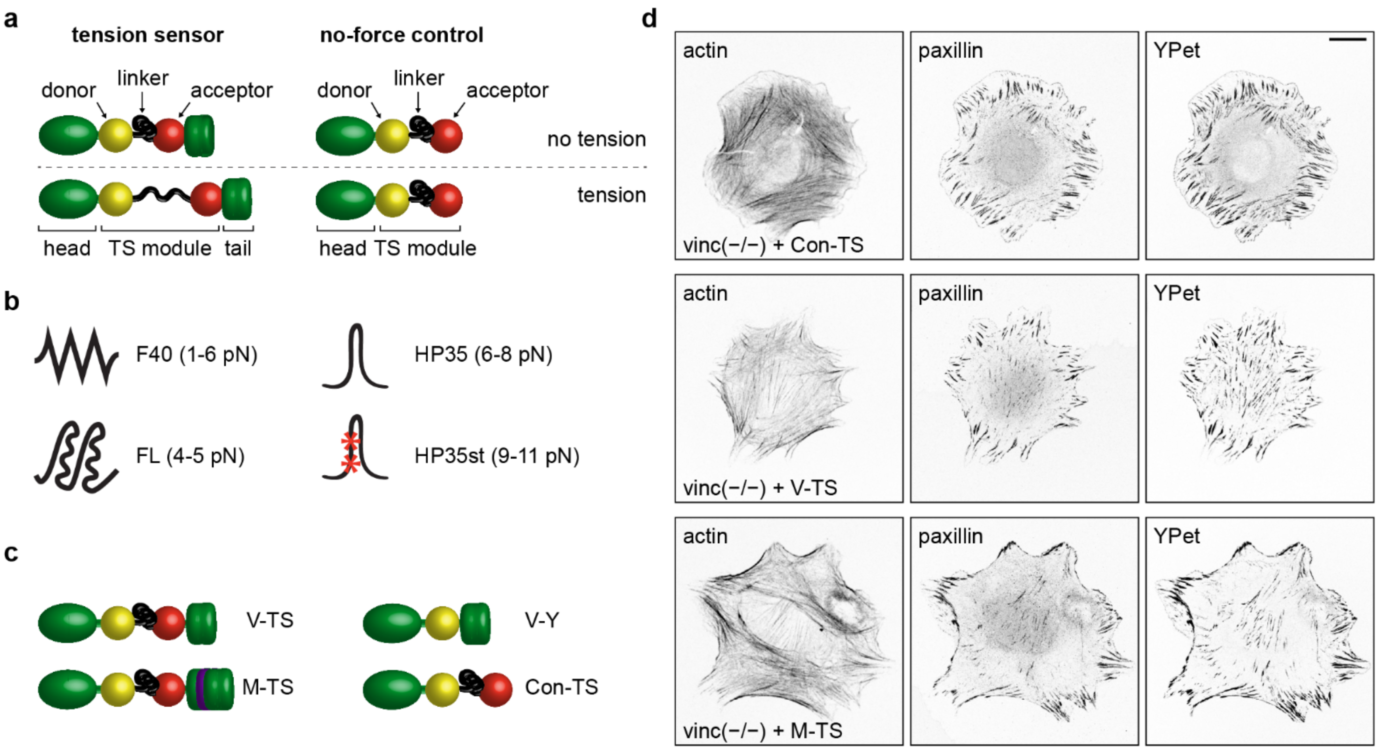

Supplementary Figure 3 Expression constructs used for vinculin isoform–specific force measurements in cells. (**a**) Schematic depiction of the tension sensor (TS) principle showing the vinculin construct as an example. The TS module, comprising donor (YPet) and acceptor (mCherry) fluorophores that are connected by a mechanosensitive linker, is integrated between head and tail region of vinculin. Mechanical force across vinculin extends the linker peptide leading to a separation of donor and acceptor fluorophore resulting in a FRET reduction. The no-force control lacks the C-terminal domain and is force insensitive. Note that domains are not drawn to scale. (**b**) Schematic depiction of force-sensitive linker peptides used for (meta)vinculin FRET-FLIM studies. Forces at the indicated ranges lead to linker peptide extension accompanied by FRET efficiency reduction. (**c**) Schematic depiction of constructs used for (meta)vinculin FRET-FLIM studies: vinculin tension sensor (V-TS) and metavinculin tension sensor (M-TS) with a TS module inserted after aa 883; donor-only-lifetime control with analogously placed YPet (V-Y); no-force control (Con-TS) comprising the head domain (1-883) and a TS module. (**d**) Representative images of vinculin-deficient (vinc$^{(-/-)}$) cells expressing Con-TS, V-TS, and M-TS. FAs were visualized with a paxillin antibody in cells spreading for 4 h on FN-coated glass slides; f-actin was labeled with phalloidin. Scale bar: 20 µm. Note that the images of YPet and paxillin are shown as merge in Fig. 2a. Elements in all schemes: YPet donor fluorophore (yellow), mCherry acceptor fluorophore (red), vinculin (green), metavinculin insert (purple), mechanosensitive linker (black).

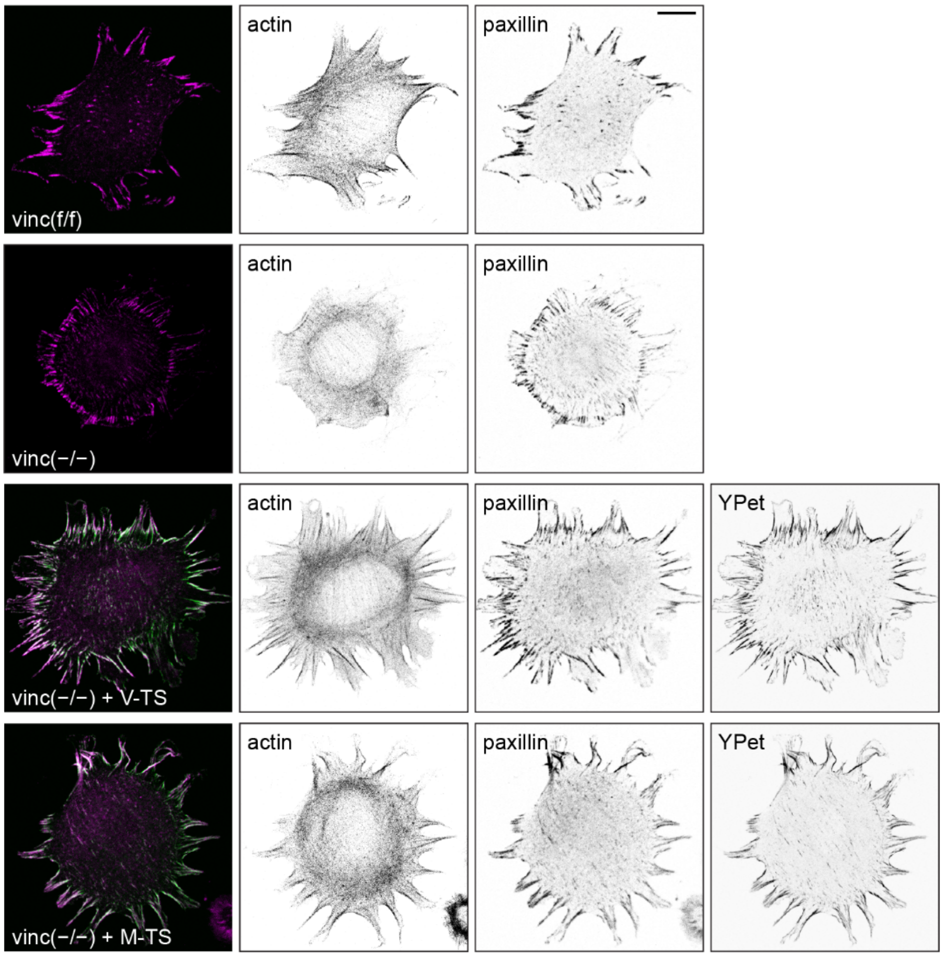

Supplementary Figure 4 Vinculin and metavinculin tension sensors rescue the spreading defect of vinculin-deficient cells. Representative images of parental (vinc$^{(f/f)}$), vinculin-deficient (vinc$^{(-/-)}$), and vinc$^{(-/-)}$ cells reconstituted with vinculin tension sensor (V-TS) and metavinculin tension sensor (M-TS) illustrate the rescue of cell morphology by the fusion proteins. FAs were visualized with a paxillin antibody in cells spreading for 2 h on FN-coated glass slides; f-actin was labeled with phalloidin. Grey scale images depict the individual channels, the merge image shows paxillin in magenta and V/M-TS in green. Scale bar: 20 µm.

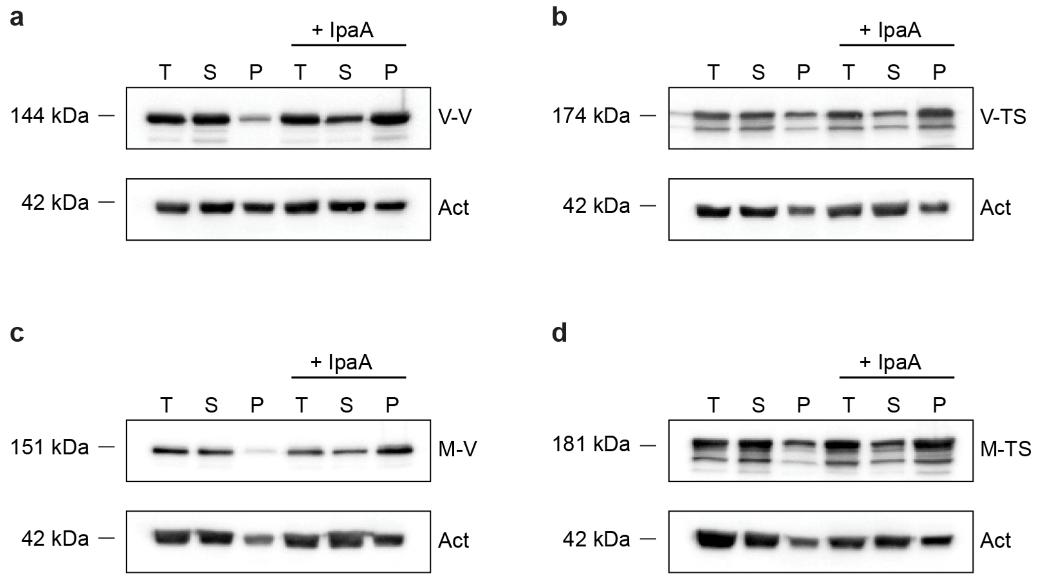

Supplementary Figure 5 Insertion of the TS module does not lead to constitutive activation of vinculin and metavinculin. Actin co-sedimentation assays using hypotonic lysates of HEK293 cells expressing **(a)** vinculin-venus (V-V), **(b)** vinculin tension sensor (V-TS), **(c)** metavinculin-venus (M-V), or **(d)** metavinculin tension sensor (M-TS) in the presence or absence of the vinculin activator IpaA confirm that TS module insertion does not lead to constitutive activation of (meta)vinculin. SDS-PAGE and Western blotting analysis used 2% of total (T) and soluble (S) fractions, and 10% of the pellet (P) fraction. Note the decrease in the soluble (S) and increase in the pellet (P) fraction upon addition of IpaA in all samples. The processing of mCherry-containing tension sensor constructs (V-TS and M-TS) for SDS-PAGE analysis leads to a partial fragmentation of the protein, as described for DsRed-derived fluorophores before[56]. Sample processing controls (Act) show that equal protein concentrations were used. Note that samples were derived from the same experiments and gels/blots were processes in parallel. Expected molecular weight values are indicated (kDa). Uncropped immunoblots with protein markers are provided in the Source Data file.

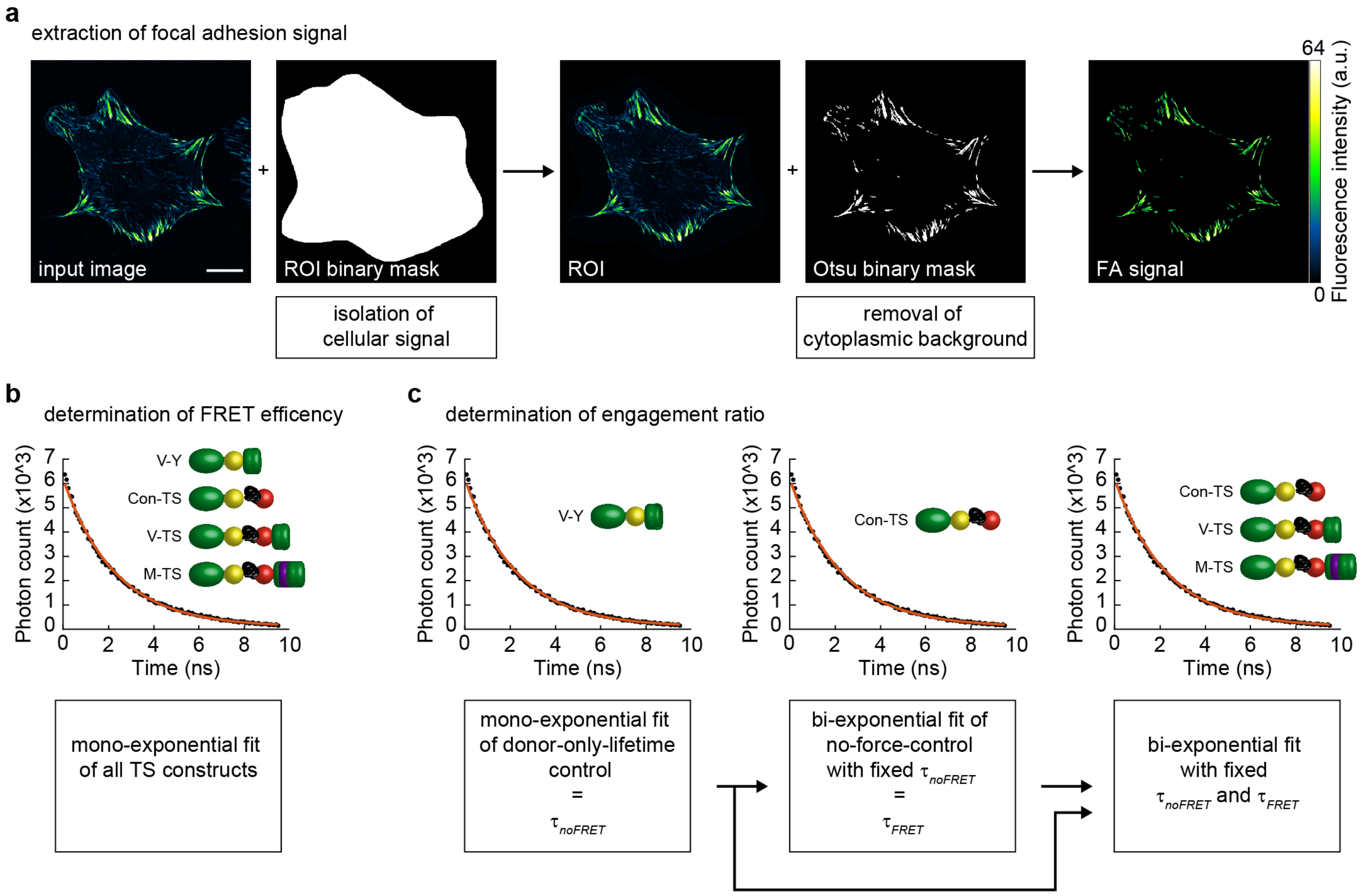

Supplementary Figure 6 Schematic depiction of FA analysis workflow of FRET-FLIM data

(a) The signal specific to FAs is extracted from an example intensity image generated by summing the temporal information of photon arrivals of the donor fluorophore from the raw FLIM data. The signal is limited to one cell only by manually drawing a region of interest (ROI) around it. To remove cytoplasmic and background signal, the FA-specific signal is isolated by an automated intensity-based multi-Otsu thresholding algorithm followed by exclusion of regions smaller than ~0.5 µm^2. Therefore, the subsequently fitted lifetimes represent an average of the lifetimes of all isolated FAs of one individual cell represented by one dot in the boxplots. Scale bar: 20 µm. **(b)** To analyze FRET efficiencies, the fluorescence lifetime of donor-only-lifetime control (V-Y), no-force control (Con-TS), vinculin tension sensor (V-TS) and metavinculin tension sensor (M-TS) are determined by fitting a mono-exponential decay function (red line) to the photon count time trace (black dots) of each masked cell. **(c)** Molecular engagement ratios are determined in a three-step process: First, τ_{noFRET} is approximated by the lifetime of the donor-only-lifetime control (V-Y) determined by mono-exponential fit as described in (b). Next, τ_{FRET} is determined as the short lifetime in a bi-exponential fit of the no-force control FLIM data. Finally, molecular engagement ratios are estimated from the FLIM data of the no-force control (Con-TS) and the tension sensors (V-TS and M-TS) fitted by a bi-exponential fit with fixed τ_{noFRET} and τ_{FRET}. The resulting ratios are rescaled to the engagement ratio of the no-force

control to correct for non-fluorescent acceptor fluorophores. Note that an example data set is depicted in each graph. The schematic drawings of protein constructs in (b) and (c) are described in detail in Supplementary Fig. 3a, c; vinculin (green), metavinculin insert (purple), YPet donor fluorophore (yellow), mCherry acceptor fluorophore (red), mechanosensitive linker (black).

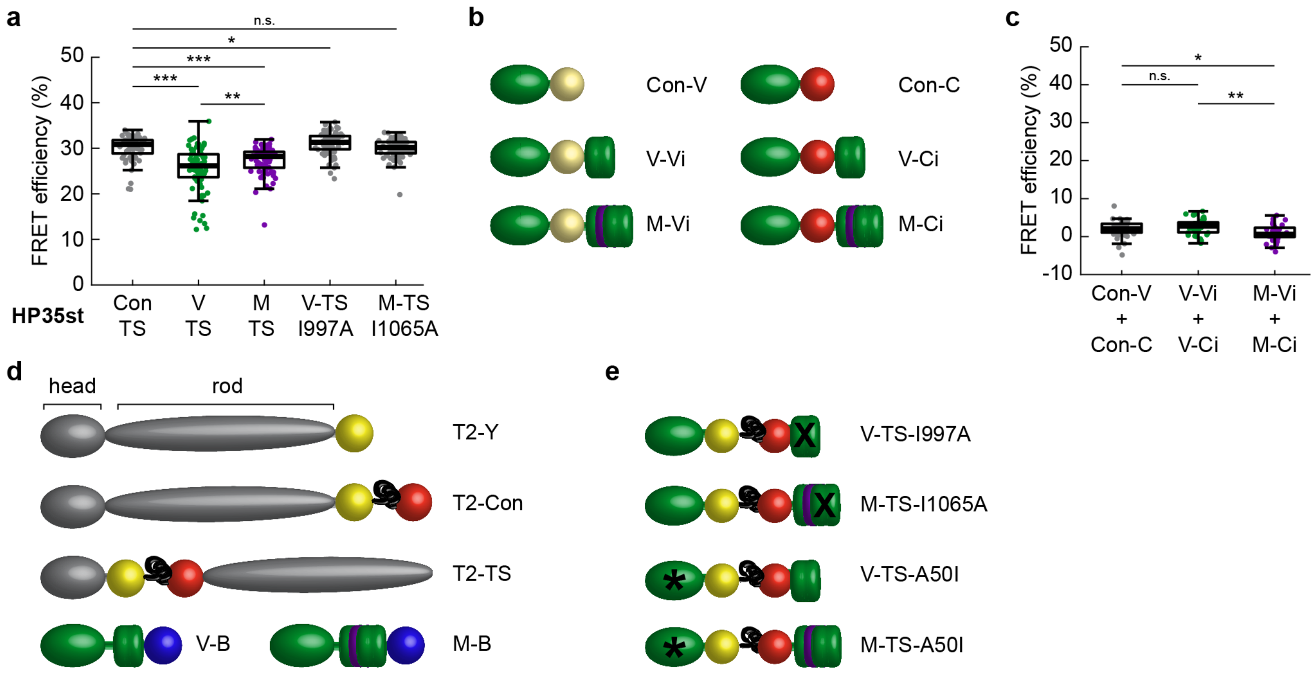

Supplementary Figure 7 Additional expression constructs and control experiments for vinculin isoform–specific force measurements. (**a**) Live-cell FLIM measurements of vinculin-deficient (vinc$^{(-/-)}$) cells expressing HP35st-based vinculin (V-TS) and metavinculin tension sensor (M-TS) reveal forces of at least 9–11 pN across both isoforms in FAs. However, FRET efficiencies in M-TS samples are higher as compared to V-TS. Actin-binding mutants of vinculin (V-TS-I997A) and metavinculin (M-TS-I1065A) tension sensors demonstrate that the observed effect is likely caused by differential actin engagement. (n = 72, 77, 77, 75, 74 cells). (**b**) To determine intermolecular FRET, the donor (venus) and acceptor (mCherry) fluorophores were integrated separately into vinculin (V-Vi, V-Ci) and metavinculin (M-Vi, M-Ci) after aa 883 or added to the C-terminus of the (meta)vinculin head domain (aa 1-883) (Con-V, Con-C). (**c**) Live-cell FLIM measurements of cells co-expressing intermolecular FRET controls reveal negligible FRET efficiencies of around 0.6–3%. Intermolecular FRET in metavinculin expressing cells is slightly lower, demonstrating that the difference in FRET efficiency between V-TS and M-TS samples is not an intermolecular FRET artefact. (n = 40, 36, 39 cells). (**d**) Schematic depiction of constructs used for FRET-FLIM analysis of talin-2 tension in the presence of vinculin or metavinculin: talin-2 (grey) C-terminally–tagged with YPet or the HP35-based TS module constitute the donor-only-lifetime (T2-Y) and the no-force (T2-Con) controls, respectively; talin-2 tension sensor (T2-TS) contains the same TS module inserted after aa 450 (between the head and tail domain); vinculin (V-B) and metavinculin (M–B) are C-terminally tagged with TagBFP (blue). (**e**) Schematic depiction of constructs used for mutational (meta)vinculin FRET-FLIM studies: the actin-binding-impaired tension sensor mutants of vinculin (V-TS-I997A) and metavinculin (M-TS-I1065A) contain a point mutation in the tail domain (X); vinculin (V-TS-A50I) and

metavinculin (M-TS-A50I) tension sensors with reduced affinity to talin contain an A50I point mutation (*) in the head domain. Elements in all schemes: YPet and venus donor fluorophores (yellow), mCherry acceptor fluorophore (red), vinculin (green), metavinculin insert (purple), mechanosensitive linker (black). Boxplots show median, 25th and 75th percentile with whiskers reaching to the last data point within 1.5× interquartile range. Two-sided Kolmogorov–Smirnov test: *** $p < 0.001$, ** $p < 0.01$, * $p < 0.05$, n.s. (not significant): $p \geq 0.05$. Source data and exact p-values are provided in the Source Data file.

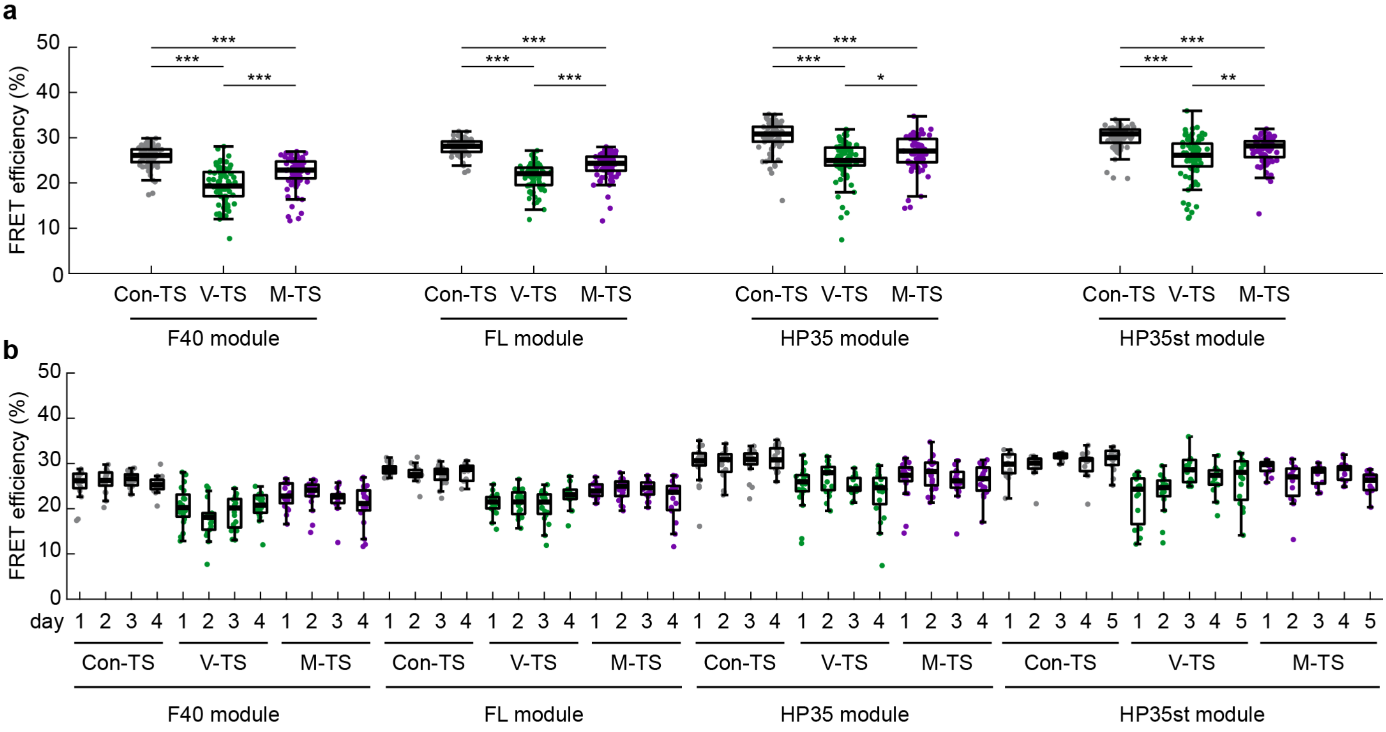

Supplementary Figure 8 Four different TS modules used to investigate vinculin isoform–specific force transduction. (**a**) Live-cell FLIM measurements of vinculin-deficient (vinc$^{(-/-)}$) cells expressing no-force control (Con-TS), vinculin tension sensor (V-TS), and metavinculin tension sensor (M-TS) constructs based on TS modules with distinct force sensitivities: F40 (1–6 pN), FL (3–5 pN), HP35 (6–8 pN), and HP35st (9–11 pN). Differences between V-TS and M-TS become smaller at higher force ranges, but they are evident in all experiments, pointing towards a reduced proportion of engaged linkages in metavinculin-expressing cells; data of FL and HP35st TS modules are the same as in Supplementary Fig. 3f and in Fig. 2c, respectively. (F40: n = 74, 77, 80 cells; FL: n = 73, 73, 74 cells; HP35: n = 78, 80, 80 cells; HP35st: n = 72, 77, 77 cells). (**b**) Plotting FRET efficiency values of individual experimental days demonstrates reproducibility of the experiments. (F40-Con-TS: n = 20, 17, 17, 20 cells; F40-V-TS: n = 20, 18, 19, 20 cells; F40-M-TS: n = 20, 22, 17, 21 cells; FL-Con-TS: n = 18, 20, 21, 14 cells; FL-V-TS: n = 18, 20, 20, 15 cells; FL-M-TS: n = 18, 21, 20, 15 cells; HP35-Con-TS: n = 20, 18, 20, 20 cells; HP35-V-TS: n = 21, 19, 20, 20 cells; HP35-M-TS: n = 21, 20, 19, 20 cells; HP35ST-Con-TS: n = 15, 16, 10, 15, 16 cells; HP35ST-V-TS: n = 15, 16, 15, 15, 16 cells; HP35ST-M-TS: n = 15, 15, 15, 17, 15 cells). Boxplots show median, 25th and 75th percentile with whiskers reaching to the last data point within 1.5× interquartile range. Two-sided Kolmogorov–Smirnov test: *** $p < 0.001$, ** $p < 0.01$, * $p < 0.05$. Source data and exact p-values are provided in the Source Data file.

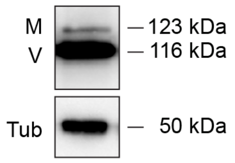

Supplementary Figure 9 HL-1 cells naturally express metavinculin. Western blot analysis of HL-1 cells, derived from mouse atrial cardiomyocytes, reveals natural expression of metavinculin (M). vinculin (V), tubulin (Tub). Expected molecular weight values are indicated (kDa); uncropped immunoblots with protein markers are provided in the Source Data file.

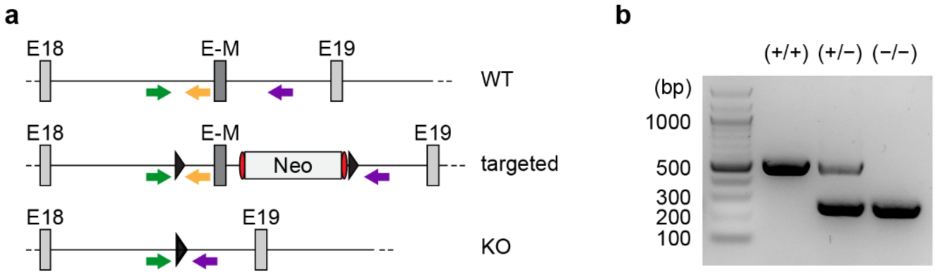

c

genotype	# of mice	ratio
(+/+)	84	0.24
(+/−)	183	0.53
(−/−)	79	0.23
total	346	1.00

Supplementary Figure 10 Generation of metavinculin-deficient mice with the homologous recombination approach. (**a**) Scheme of the targeting strategy used to remove the exon encoding for the metavinculin insert (E-M) from the *Vcl* gene (WT). The E-M exon was flanked by loxP sites (triangles) in the targeting construct. A neomycin-resistance cassette (Neo) was included to facilitate selection of modified ES cells, carrying the targeted allele, which were used to generate chimeric animals. Subsequent breeding with Cre transgenic mice led to excision of the fragment between loxP sites, resulting in the knockout allele (KO). (**b**) Representative results of the genotyping PCR showing examples of a wild type (+/+), heterozygous (+/−), and knockout (−/−) sample. The three-primer PCR (see (a)) gives rise to the bigger fragment (504 bp) on a WT allele template (green and yellow primer) and a smaller fragment (245 bp) on a KO allele template (green and purple primer). The large fragment, potentially generated on a WT allele template with green/purple primer pair, is not visible due to short extension step of the PCR reaction. (**c**) Heterozygous mice breed at Mendelian ratio ($X^2 = 1.301$; $p = 0.52$; $X^2_{0.05} = 5.991$). Source data are provided in the Source Data file.

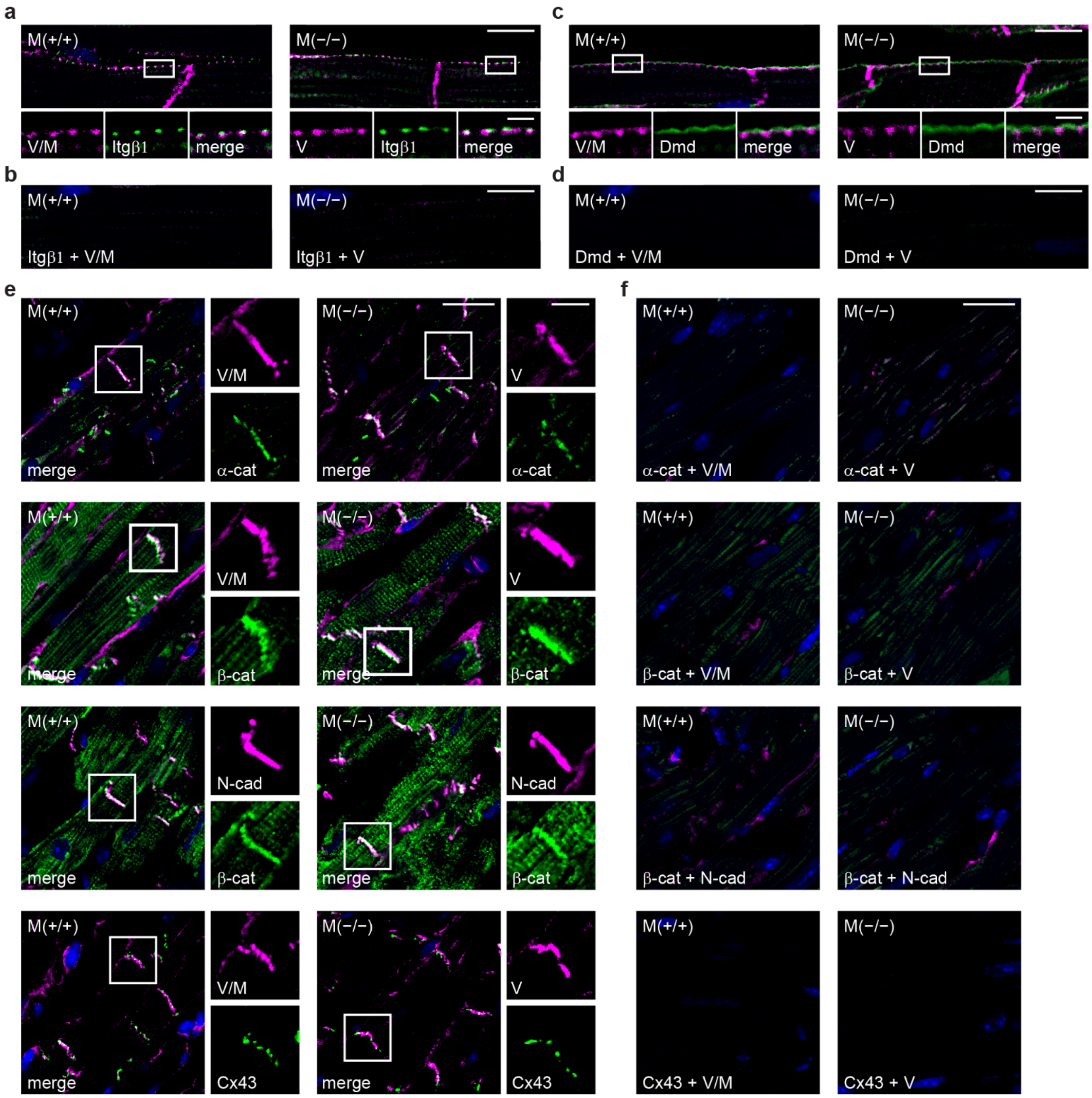

Supplementary Figure 11 Normal tissue architecture in the absence of metavinculin. (**a**) Representative immunohistochemistry of ventricular tissue from 13-month-old wild type $M^{(+/+)}$ and metavinculin knockout $M^{(-/-)}$ mice showing β1 integrin (Itgβ1) to visualize costameres. (**b**) Control staining using only secondary antibodies confirms the specificity of signal observed in (a). (**c**) Immunohistochemistry of ventricular tissue from 13-month-old $M^{(+/+)}$ and $M^{(-/-)}$ mice showing dystrophin localization (Dmd). (**d**) Control staining using only secondary antibodies confirms the specificity of the signal observed in (c). (**e**) Immunofluorescence staining of adherens junction markers α-catenin (α-cat), β-catenin (β-cat), N-cadherin (N-cad), and a gap junction marker connexin 43 (Cx43)

in ventricular tissue of 13-month-old $M^{(+/+)}$ and $M^{(-/-)}$ mice reveal no apparent differences in tissue structure in the absence of metavinculin. The image of β-catenin–stained samples is also shown in Fig. 4c, d. (**f**) Respective control stainings using only secondary antibodies confirm the specificity of the signals shown in (e). Tissues are co-stained for (meta)vinculin (V/M) as indicated; nuclei are visualized with DAPI (blue). Scale bar: 20 µm, in zoom: 10 µm.

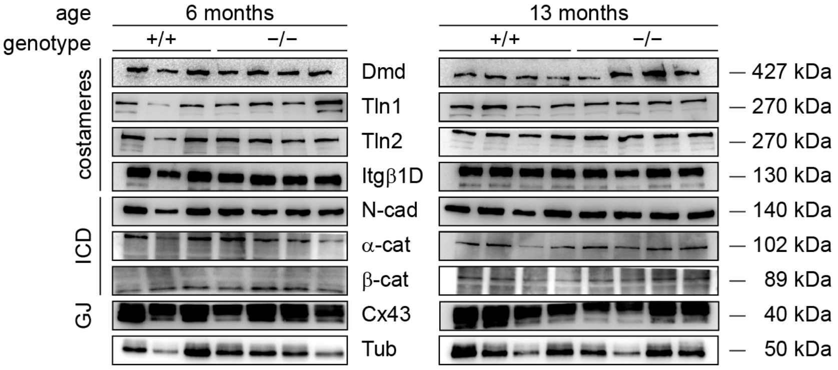

Supplementary Figure 12 Unaltered protein expression of marker proteins in metavinculin-deficient mice. Western blot analysis of ventricular tissue lysates from 6- and 13-month-old wild type $M^{(+/+)}$ and metavinculin knockout $M^{(-/-)}$ mice confirms virtually unaltered expression levels of costamere proteins (Dmd: dystrophin; Tln1: talin-1; Tln2: talin-2; Itgβ1D: integrin β1D), intercalated disc (ICD) markers (N-cad: N-cadherin; α-cat: α-catenin; β-cat: β-catenin), and the gap junction (GJ) protein connexin-43 (Cx43). Tubulin (Tub) was used as a loading control. We note that we observed degradation bands in two of the Itgβ1D samples from 6-month-old KO mice, which are presumably an artefact of the sample preparation (shown in the source data). Sample processing controls (Tub) show that equal protein concentrations were used. Note that samples were derived from the same experiments and gels/blots were processes in parallel. Expected molecular weight values are indicated (kDa). Uncropped immunoblots with protein markers are provided in the Source Data file.

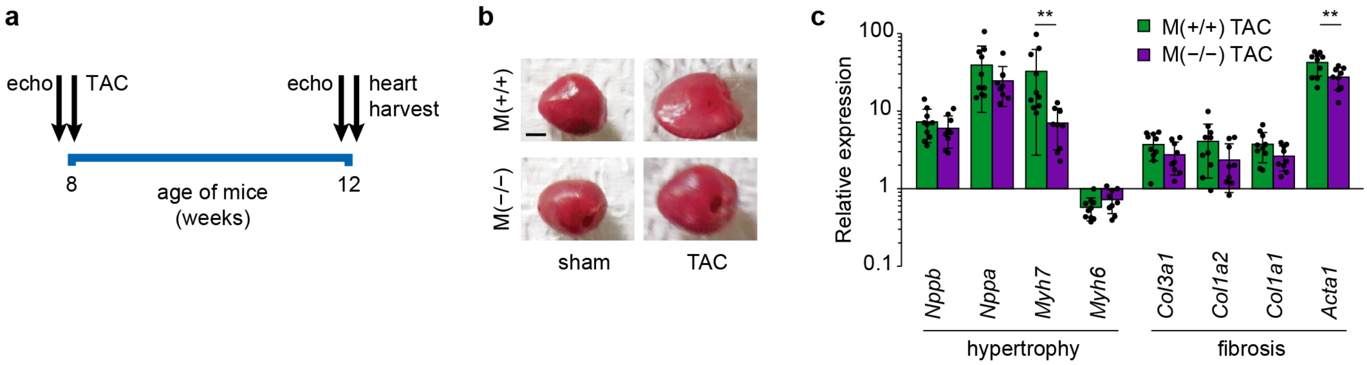

Supplementary Figure 13 Transverse aortic constriction (TAC) protocol used to induce cardiac hypertrophy in this study. (**a**) Time line of the TAC protocol. Twenty wild type $M^{(+/+)}$ and twenty metavinculin knockout $M^{(-/-)}$ 8-week-old male mice were examined by echocardiographic analysis before the experiment. Ten animals of each genotype were sham-operated and ten subjected to TAC. After four weeks, the mice were analyzed again by echocardiography and sacrificed for tissue isolation. (**b**) Representative images of isolated ventricles of sham- and TAC-operated 3-month-old $M^{(+/+)}$ and $M^{(-/-)}$ mice. Scale bar: 0.2 cm. (**c**) Real-time qPCR analysis of ventricular tissue from $M^{(+/+)}$ and $M^{(-/-)}$ mice was used to monitor cardiac remodeling. Natriuretic peptides B precursor (*Nppb*), Natriuretic peptides A precursor (*Nppa*), myosin heavy chain 7 (*Myh7*), myosin heavy chain 6 (*Myh6*), collagen type III alpha 1 chain (*Col3a1*), collagen type I alpha 2 chain (*Col1a2*), collagen type I alpha 1 chain (*Col1a1*), actin alpha 1 (*Acta1*). ($n = 10$, 9 mice). Bar chart shows mean values ± SD. Two-sided ANOVA followed by Sidak's test: ** $p < 0.01$. Source data and exact p-values are provided in the Source Data file.

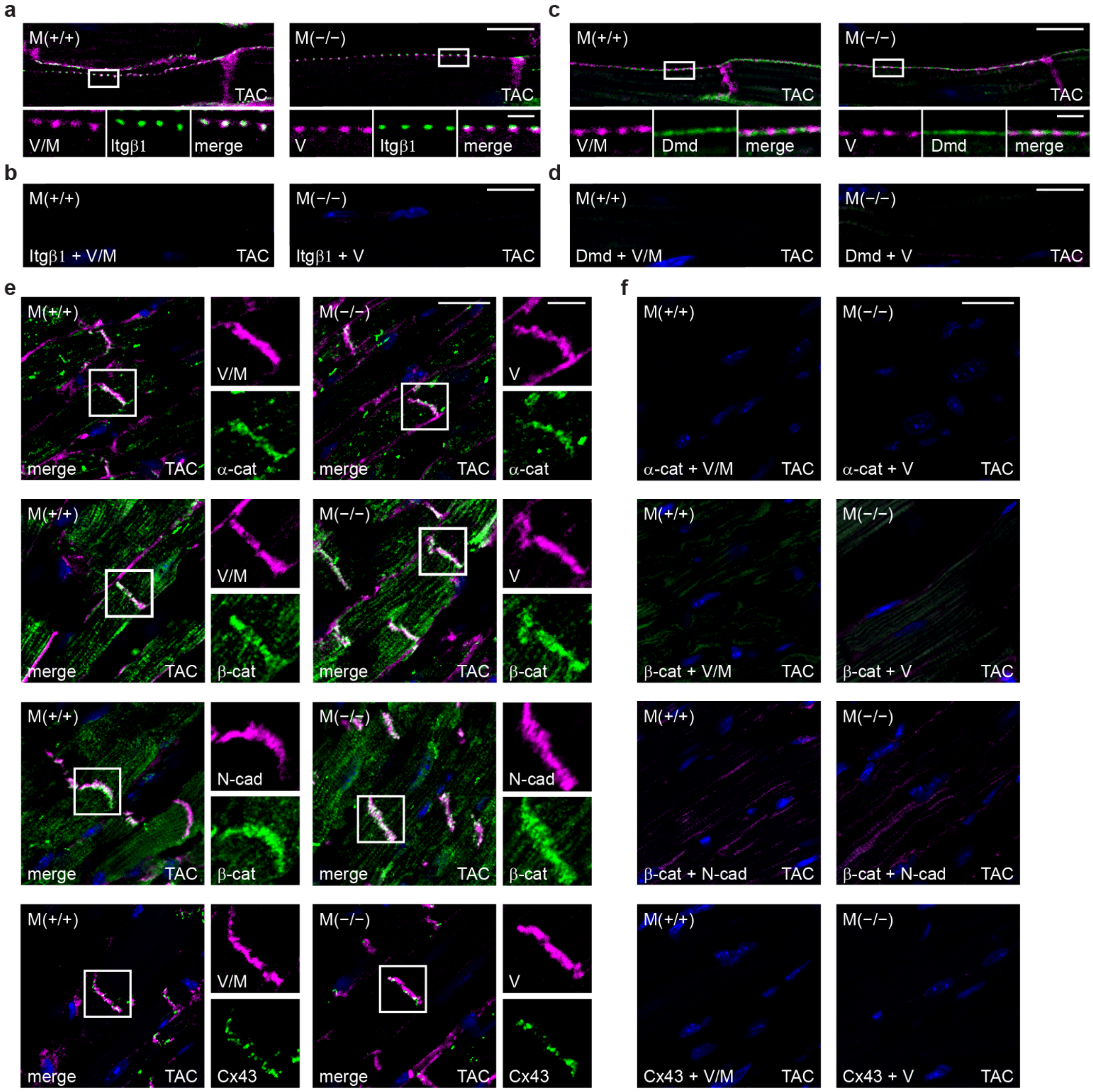

Supplementary Figure 14 The absence of metavinculin does not aggravate the hypertrophic cardiac response. (**a**) Representative immunohistochemistry of ventricular tissue from 3-month-old wild type $M^{(+/+)}$ and metavinculin knockout $M^{(-/-)}$ mice subjected to transverse aortic constriction (TAC) showing β1 integrin (Itgβ1) as marker for costameres. The image is also shown in Fig. 4i, j. (**b**) Control staining using only secondary antibodies confirms the specificity of the signal observed in (a). (**c**) Immunohistochemistry of ventricular tissue from 3-month-old $M^{(+/+)}$ and $M^{(-/-)}$ mice after TAC showing dystrophin localization. (**d**) Control staining using only secondary antibodies confirms the specificity of the observed signals in (c). (**e**) Immunofluorescence staining of adherens junction markers

α-catenin (α-cat), β-catenin (β-cat), N-cadherin (N-cad), and a gap junction marker connexin 43 (Cx43) in ventricular tissue of 3-month-old $M^{(+/+)}$ and $M^{(-/-)}$ mice after TAC revealing no apparent differences in tissue structure in the absence of metavinculin. The image of β-catenin–stained samples is also shown in Fig. 4k, l. (**f**) Respective control stainings using only secondary antibodies confirm the specificity of the signals shown in (e). Tissues are co-stained for (meta)vinculin (V/M) as indicated; nuclei are visualized with DAPI (blue). Scale bar: 20 µm, in zoom: 10 µm.

Supplementary tables

	before surgery				after surgery			
Treatment	sham	sham	TAC	TAC	sham	sham	TAC	TAC
genotype	M(+/+)	M(−/−)	M(+/+)	M(−/−)	M(+/+)	M(−/−)	M(+/+)	M(−/−)
number	10	9	9	7	10	9	9	7
HR [BPM]	436.92 ± 57.90	471.38 ± 49.20	469.76 ± 52.35	432.35 ± 53.77	435.86 ± 90.87	420.66 ± 59.55	427.59 ± 86.55	465.26 ± 45.99
SV [µl]	27.38 ± 4.89	26.21 ± 7.69	26.29 ± 3.15	27.25 ± 3.74	27.97 ± 5.80	29.25 ± 7.72	15.79 ± 4.14 ***	14.88 ± 3.26 ***
EF [%]	47.14 ± 5.83	50.26 ± 3.39	50.02 ± 4.38	50.72 ± 5.39	46.54 ± 3.89	48.02 ± 2.45	20.54 ± 4.88 ***	20.66 ± 5.89 ***
FS [%]	23.19 ± 3.34	24.90 ± 2.00	24.80 ± 2.52	25.27 ± 3.20	22.81 ± 2.14	23.68 ± 1.33	9.24 ± 2.34 ***	9.29 ± 2.74 ***
CO [ml/min]	12.15 ± 3.57	12.31 ± 4.10	12.31 ± 1.78	11.72 ± 1.70	11.75 ± 1.95	11.76 ± 2.09	6.65 ± 2.02 ***	6.94 ± 1.71 **

Supplementary Table 1 Echocardiography parameters measured before and four weeks after performing TAC surgery.

Comparison of heart rate (HR), stroke volume (SV), ejection fraction (EF), fractional shortening (FS) and cardiac output (CO) values of wild type $M^{(+/+)}$ and metavinculin knockout $M^{(-/-)}$ mice before and four weeks after sham or transverse aortic constriction (TAC) surgery. No significant differences were observed between $M^{(-/-)}$ and $M^{(+/+)}$ mice before or after undergoing TAC procedure. *** $p < 0.001$, ** $p < 0.01$, * $p < 0.05$. P-values for evaluating parameters "before surgery" and "after surgery" were calculated by two-sided ANOVA followed by Sidak's multiple comparison test. Data represent means ± SD. Source data are provided in the Source Data file.

Supplementary Table 2 List of RT-qPCR primers

gene	forward primer	reverse primer
Nppa mouse	5′-GCTTCCAGGCCATATTGGAG-3′	5′-GGGGGCATGACCTCATCTT-3′
Nppb mouse	5′-CCCAAAAAGAGTCCTTCGGTC-3′	5′-CGGTCTATCTTGTGCCCAAAG-3′
Myh6 mouse	5′-GCCCAGTACCTCCGAAAGTC-3′	5′-GCCTTAACATACTCCTCCTTGTC-3′
Myh7 mouse	5′-ACTGTCAACACTAAGAGGGTCA-3′	5′-TTGGATGATTTGATCTTCCAGGG-3′
Acta1 mouse	5′-CCCAAAGCTAACCGGGAGAAG-3′	5′-CCAGAATCCAACACGATGCC-3′
Col1a1 mouse	5′-CTGGCAAGAAGGGAGATGA-3′	5′-CACCATCCAAACCACTGAAA-3′
Col1a2 mouse	5′-AGGTCTTCCTGGAGCTGATG-3′	5′-ACCCACAGGGCCTTCTTTAC-3′
Col3a1 mouse	5′-ACAGCAAATTCACTTACACAGTTC-3′	5′-CTCATTGCCTTGCGTGTTT-3′
RPL32 mouse	5′-ACATCGGTTATGGGAGCAAC-3′	5′-GGGATTGGTGACTCTGATGG-3′
