# Supplementary material for: Metavinculin modulates force transduction in cell adhesion sites
Source: Nat Commun. 2020 Dec 17;11:6403. doi: 10.1038/s41467-020-20125-z (PMC7747745; doi:10.1038/s41467-020-20125-z)
Supplement: Supplementary file 3 — Reporting Summary [file 41467_2020_20125_MOESM3_ESM.pdf]

nature research

Corresponding author(s): Carsten Grashoff

Last updated by author(s): Nov 13, 2020

Reporting Summary

Nature Research wishes to improve the reproducibility of the work that we publish. This form provides structure for consistency and transparency in reporting. For further information on Nature Research policies, see Authors & Referees and the Editorial Policy Checklist.

Statistics

For all statistical analyses, confirm that the following items are present in the figure legend, table legend, main text, or Methods section.

n/a	Confirmed	
☐	☒	The exact sample size (*n*) for each experimental group/condition, given as a discrete number and unit of measurement
☐	☒	A statement on whether measurements were taken from distinct samples or whether the same sample was measured repeatedly
☐	☒	The statistical test(s) used AND whether they are one- or two-sided<br>*Only common tests should be described solely by name; describe more complex techniques in the Methods section.*
☒	☐	A description of all covariates tested
☐	☒	A description of any assumptions or corrections, such as tests of normality and adjustment for multiple comparisons
☐	☒	A full description of the statistical parameters including central tendency (e.g. means) or other basic estimates (e.g. regression coefficient) AND variation (e.g. standard deviation) or associated estimates of uncertainty (e.g. confidence intervals)
☐	☒	For null hypothesis testing, the test statistic (e.g. *F*, *t*, *r*) with confidence intervals, effect sizes, degrees of freedom and *P* value noted<br>*Give P values as exact values whenever suitable.*
☒	☐	For Bayesian analysis, information on the choice of priors and Markov chain Monte Carlo settings
☒	☐	For hierarchical and complex designs, identification of the appropriate level for tests and full reporting of outcomes
☒	☐	Estimates of effect sizes (e.g. Cohen's *d*, Pearson's *r*), indicating how they were calculated

Our web collection on statistics for biologists contains articles on many of the points above.

Software and code

Policy information about availability of computer code

Data collection

For confocal imaging on Leica TCS SP5 X and Zeiss LSM 880 the Leica Application Suite Advanced Fluorescence (version 2.7.3.9723) and the ZEN Software (black edition) were used respectively. Confocal images of tissues were acquired on a LSM 780 using the ZEN 2.1 software (version 11.0, Zeiss). All confocal images were processed in FiJi.
For acquiring FLIM data on the Leica TCS SP5 X system the Leica Application Suite Advanced Fluorescence (version 2.7.3.9723) was used together with Imspector Pro (LaVision).
For acquiring FLIM data on the Zeiss LSM 880 system the ZEN Software (black edition) was used together with the SymPhoTime 64 software (PicoQuant).
FRAP data was collected on a Leica SP8 confocal laser scanning microscope using LAS X Software (version 3.5.5.19976).
Histological images were acquired on a Zeiss Observer Z1 using MetaMorph 7.7.1.0 or on a Axioskop (Zeiss) using SPOT v5.1.
Pulse-wave Doppler echocardiography was perforemd on a Vevo Imaging System (Fujifilm Visual Sonics) using VevoLab.
qPCR data was acquired on Light Cycler 480 II (Roche) using LightCycler 480 Software (version 1.5).

Data analysis

FLIM data and cellular eccentricity was analyzed using custom-written MATLAB software, which is avialbale on request.
Two-sided Kolmogorov–Smirnov (KS) statistical tests were performed in MATLAB R2017b. Two-sided analysis of variance (ANOVA) followed by Sidak's multiple comparison test was performed on GraphPad Prism v6.0.
Quantification of interstitial fibrosis was performed using MetaMorph 7.7.1.0., FRAP analysis with the Fiji plug-in FRAP profiler (http://worms.zoology.wisc.edu/ImageJ/FRAP_Profiler.java) and custom-written MATLAB softwear (MATLABR2017b). Analysis of qPCR data was performed with Excel Office 2019.

For manuscripts utilizing custom algorithms or software that are central to the research but not yet described in published literature, software must be made available to editors/reviewers. We strongly encourage code deposition in a community repository (e.g. GitHub). See the Nature Research guidelines for submitting code & software for further information.

Data

Policy information about availability of data

All manuscripts must include a data availability statement. This statement should provide the following information, where applicable:

- Accession codes, unique identifiers, or web links for publicly available datasets
- A list of figures that have associated raw data
- A description of any restrictions on data availability

The authors confirm that all relevant data are included in this published article (and its supplementary information files). Additional data is available upon request.

Field-specific reporting

Please select the one below that is the best fit for your research. If you are not sure, read the appropriate sections before making your selection.

☒ Life sciences ☐ Behavioural & social sciences ☐ Ecological, evolutionary & environmental sciences

For a reference copy of the document with all sections, see nature.com/documents/nr-reporting-summary-flat.pdf

Life sciences study design

All studies must disclose on these points even when the disclosure is negative.

Sample size	Shown immunostainings and live cell images are representative of at least 2-3 independent experiments. In case of FLIM experiments, at least 30-80 individual cells were recorded and, to ensure reproducibility, experiments were recorded over 2–6 independent days. Transverse aortic constriction was performed on 10 mice per operation- and genotype. All IF and histological analysis on heart tissue sections was performed on at least 3 different regions of at least 3 different mice per condition. Formal sample size calculation was not performed. Instead, sample size for FLIM experiments was based on previous publications (Austen et al. NCB, 2015; Ringer et al. Nature Methods; 2017 and Price et al. Nat Comm, 2019) showing that biological effects can be documented and statistically evaluated with the chosen number of cells. Sample size was kept similar between experimental conditions. In agreement with the ethics oversight committee (Government of Upper Bavaria, Germany) and the collaborating laboratory at the TU Munich (Engelhardt lab), the minimal number of mice, required to document statistic significant effects, was used.
Data exclusions	FLIM data analysis was automatized and restricted to the focal adhesion or cell-cell contact signal as described in Materials and Methods. Images with insufficient signal intensity were excluded manually from the analysis.<br>Echocardiographic data of mice dying during the second echocardiography were excluded. In qPCR analysis Ct values of triplicates differing by more than 0.5 were excluded. All data exclusion criteria were pre-established.
Replication	All cell-experiments were repeated independently on at least two independent experimental days. Typically, FLIM experiments were repeated over 2–6 days recording 20-30 images on each day per construct and condition; an example for the reproducibility of FLIM results across individual days is shown in Supplementary Figure 4.<br>All mouse experiments were performed on at least 3 different mice per condition. Immunofluorescence and histological analysis on heart tissue sections was performed on at least 3 different regions of each mouse.<br>All replication attempts were successful and in the expected variability range.
Randomization	In cell-experiments, the groups were determined by the transfected construct and all experiments were started from the same batch of vcl (-/-) cells. In mouse-experiments, groups were determined by genotype, while mice of the same age and generation were compared. No further particular randomization strategy was implemented.<br>For TAC-experiment, randomization was ensured by allocating every second littermate to sham or TAC operation group.
Blinding	For cell data acquisition and analysis the investigators were not blinded, since transiently transfected cells have very different expression levels and cells with expression levels similar to endogenous levels were chosen. Furthermore, the phenotype of cells expressing the different constructs is in many cases obvious and makes blinding impossible. However, all FLIM data analysis were subjected to the same automated analysis procedure.<br>Blinding was not feasible for mouse experiments, because age influences heart conditions. Therefore, mice of the same age and from the same generation were compared. The experimenters performing TAC operation as well as organ harvest were blinded to the genotypes.

Reporting for specific materials, systems and methods

We require information from authors about some types of materials, experimental systems and methods used in many studies. Here, indicate whether each material, system or method listed is relevant to your study. If you are not sure if a list item applies to your research, read the appropriate section before selecting a response.

Materials & experimental systems

n/a	Involved in the study	
☐	☒	Antibodies
☐	☒	Eukaryotic cell lines
☒	☐	Palaeontology
☐	☒	Animals and other organisms
☒	☐	Human research participants
☒	☐	Clinical data

Methods

n/a	Involved in the study	
☒	☐	ChIP-seq
☒	☐	Flow cytometry
☒	☐	MRI-based neuroimaging

Antibodies

Antibodies used

mouse anti-actin (sarcomeric) (Sigma, A2172)
rabbit anti-α-catenin (Sigma, C2081)
rabbit anti-β-catenin (Sigma, C2206)
rabbit anti-connexin 43 (Cell Signaling Technologies, 3512)
rabbit anti-dystrophin (Abcam, ab15277)
rat anti-integrin β1 (MB1.2, Millipore, MAB1997)
mouse anti-integrin β1d (2B1, abcam, ab8991)
rabbit anti-FAK (Millipore, 06-543)
rabbit anti-ILK (Cell Signaling Technologies, 3862)
mouse anti-GFP (Sigma, G1546)
mouse anti-N-cadherin (3B9, Thermo Fisher Scientific, 33-3900)
mouse anti-paxillin (BD Transduction Laboratories, 610051/2)
mouse anti-talin 1 (97H6, Bio-Rad, MCA4770)
mouse anti-talin 2 (68E7, abcam, ab105458)
mouse anti-tubulin (DM1A, Sigma, T6199)
mouse anti-vinculin (hVIN-1, Sigma, C2081)
anti-mouse IgG Alexa Fluor-405 (Invitrogen, A31553)
anti-rabbit IgG Alexa Fluor-405 (Invitrogen, A31556)
anti-rabbit IgG Alexa Fluor-488 (Invitrogen, A21441)
anti-rat IgG Alexa Fluor-488 (Invitrogen, A11006)
anti-mouse IgG Alexa Fluor-568 (Invitrogen, A11004)
anti-rabbit IgG Alexa Fluor-568 (Invitrogen, A11036)
anti-mouse IgG Alexa Fluor-647 (Invitrogen, A21235)
anti-mouse IgG HRP (BioRad, 170-6516)
anti-rabbit IgG HRP (BioRad, 170-6515)

Validation

mouse anti-actin (sarcomeric) (monoclonal 5C5, Sigma, A2172; WB 1:1000):
application(s): immunohistochemistry (formalin-fixed, paraffin-embedded sections): 1:500 using human skeletal and cardiac muscle, immunohistochemistry (frozen sections): suitable using human skeletal and cardiac muscle, indirect ELISA: suitable, Western blot: suitable
species reactivity: carp, rat, bovine, sheep, snake, frog, guinea pig, human, rabbit

rabbit anti-α-catenin (polyclonal, Sigma, C2081; WB: 1:4000)
application(s): dot blot: suitable using α-catenin peptide amino acids 890-901 conjugated to BSA; immunohistochemistry (frozen sections): 1:2000 using bovine kidney sections; indirect immunofluorescence: 1:2000 using cultured MDBK cells; microarray: suitable; Western blot: 1:4000 using cultured MDBK cells extract
species reactivity: several mammalian species
specificity: does not cross-react with β-catenin or γ-catenin (plakoglobin).

rabbit anti-β-catenin (polyclonal, Sigma, C2206; WB: 1:4000)
application(s): dot blot: suitable using the immunogen and related peptides conjugated to BSA; immunocytochemistry: 1:2000 using cultured MDBK cells; immunohistochemistry: 1:2000 using bovine kidney frozen sections; Western blot: 1:4000 using cultured MDBK cells
species reactivity: several mammalian species
specificity: the antibody does not cross react with α-catenin or γ-catenin (plakoglobin).

rabbit anti-connexin 43 (polyclonal, Cell Signaling Technologies, 3512; IF: 1:400, WB: 1:4000)
application(s): Western blotting (1:1000), immunohistochemistry (paraffin) (1:100), immunofluorescence (frozen) (1:50 - 1:100), immunofluorescence (immunocytochemistry) (1:50 - 1:100)
species reactivity: human, mouse, rat, monkey, zebrafish, predicted: dog and pig
specificity: connexin 43 antibody detects endogenous levels of total connexin 43. This antibody does not cross-react with other connexins.

rabbit anti-dystrophin (polyclonal, Abcam, ab15277; IF: 1:200, WB: 1:500)
application(s): immunohistochemistry frozen sections (1:400) use with acetone-fixed tissues, immunohistochemistry Formalin/PFA-fixed paraffin-embedded sections (1:100).
species reactivity: dog, human, predicted mouse, rat and pig

rat anti-integrin β1 (monoclonal MB1.2, Millipore, MAB1997; IF: 1:400)
application(s): immunoprecipitation: a previous lot of this anitbody was used to immunoprecipitate VLA (β1) integrins from lysate of 106 cell equivalent: 2 μg; Flow cytometry: A 10 μg/mL concentration of a previous lot was used in FC; immunohistochemistry in frozen tissue sections: 20 μg/mL from a previous lot was used. As a result of the wide tissue distribution of VLA (β1), binding of MAB1997 to diverse cell types is commonly observed. Does not block VLA integrin mediated cell adhesion to matrix proteins. Activation of VLA (β1) integrin signaling properties by MAB1997 has not been characterized. Western blot: 10 μg/mL. Effective for samples that have been SDS-denatured and heated. Not effective for reduced samples.
species reactivity: mouse, rat, human. Expected to react with rat based on sequence homology.
specificity: reacts with β1 subunit of VLA (β1) integrins

mouse anti-integrin β1d (monoclonal 2B1, abcam, ab8991; WB: 1:1000)
application(s): immunocytochemistry (1:25-1:200), Western blot (1:100-1:1000)
species reactivity: mouse, dog, human, pig
specificity: recognizes specifically the cytoplasmic domain of integrin subunit beta 1D present in cardiac and skeletal muscle. A broad species reactivity is expected because of the conserved nature of the epitope.

rabbit anti-FAK (polyclonal, Millipore, 06-543; WB: 1:1000)
application(s): Immunocytochemistry: 10 μg/mL of a previous lot showed positive immunostaining for FAK in cells fixed with 3.7% formaldehyde; immunoprecipitation: 4 μg of a previous lot immunoprecipitated FAK from 500 μg of murine 3T3/A31 RIPA lysate.
species reactivity: human, mouse, rat, hamster, non-reactive to avian.

rabbit anti-ILK (polyclonal, Cell Signaling Technologies, 3862; WB: 1:1000)
application(s): Western blotting (1:1000); immunohistochemistry (paraffin) (1:100)
species reactivity: human, mouse, rat, monkey, bovine
specificity: ILK1 antibody detects endogenous levels of total ILK1 protein. The antibody may cross-react with ILK2.

mouse anti-GFP (monoclonal GSN149, Sigma, G1546; WB:1:1000)
application(s): indirect ELISA: suitable; Western blot: 1-2 μg/mL using extracts of cells expressing GFP fusion proteins; immunocytochemistry, immunofluorescence – cell culture cells, immunoprecipitation, Western blotting, Western blotting following immunoprecipitation.
specificity: the antibody reacts specifically with GFP-fusion proteins.

mouse anti-N-cadherin (monoclonal 3B9, Thermo Fisher Scientific, 33-3900; IF: 1:500, WB: 1:2000)
application(s): immunocytochemistry (ICC) (1-3 μg/mL), immunofluorescence (IF) (1-3 μg/mL), immunohistochemistry (IHC), immunohistochemistry (paraffin) (IHC (P)) (1-5 μg/mL), immunoprecipitation (IP) (3-5 μg), Western blot (WB) (0.5-1 μg/mL). For IHC with this antibody use formalin-fixed, paraffin-embedded tissue and heat-induced epitope retrieval.
species reactivity: chicken, human, mouse, pig, rat; published species: artificial control, dog, hamster, horse, human, mouse, non-human primate, rabbit, rat, tag, zebrafish
specificity: This antibody is specific for N-cadherin and does not cross-react with other cadherin family members including P- and E-cadherins.

mouse anti-paxillin (monoclonal 349, BD Transduction Laboratories, 610051; IF: 1:200–400)
application(s): Western blot (routinely tested), immunoprecipitation, immunohistochemistry, immunofluorescence (tested during development)
species reactivity: human (QC testing), mouse, rat, dog, chicken (tested in development)

mouse anti-talin 1 (monoclonal 97H6, Bio-Rad, MCA4770; WB: 1:1000)
application(s): immunofluorescence (methanol treatment is required prior to antibody staining), immunohistology – paraffin (1:25-1:200), this product requires antigen retrieval using heat treatment prior to staining of paraffin sections. Sodium citrate buffer pH 6.0 is recommended for this purpose), immunoprecipitation, Western blotting
species reactivity: human, mouse, rabbit.

mouse anti-talin 2 (monoclonal 68E7, abcam, ab105458; WB: 1:2000)
application(s): immunohistochemistry – frozen sections (use at an assay dependent concentration), immunocytochemistry/ immunofluorescence (1:100 - 1:1000), immunoprecipitation (1:100 - 1:1000), Western blotting (1:100 - 1:1000. Predicted molecular weight: 272 kDa), ELISA (1:100 - 1:1000).
species reactivity: mouse

mouse anti-tubulin (monoclonal DM1A, Sigma, T6199; WB: 1:3000)
application(s): immunocytochemistry: 0.5-1 μg/mL using cultured chicken fibroblasts (CFB); immunohistochemistry: suitable; immunoprecipitation (IP): suitable; microarray: suitable; Western blot: 0.5-1 μg/mL using total tissue extract from chicken gizzard.
species reactivity: bovine, rat, yeast, human, mouse, chicken, fungi, amphibian
specificity: Anti-α-tubulin antibody, mouse monoclonal recognizes an epitope located at the C-terminal end of the α-tubulin isoform (amino acids 426-430) in a variety of organisms (e.g., human, bovine, mouse, and chicken). The antibody is specific for α-tubulin in immunoblotting assays and may be used for localization of α-tubulin in cultured cells or tissue sections. The antibody reacts best with chicken fibroblasts.

mouse anti-vinculin (monoclonal hVIN-1, Sigma, V9131; IF: 1:400, WB: 1:4000)
applications(s): immunohistochemistry (frozen sections): suitable; indirect immunofluorescence: 1:400 using cultured human fibroblasts; Western blot: 1:200 using extract of human fibroblasts
species reactivity: bovine, canine, mouse, rat, turkey, human, chicken, frog

specificity: specifically labels vinculin at cell-cell and cell-substrate contacts. Reacts strongly with human vinculin. Shows cross-reactivity with smooth muscle metavinculin.
In this manuscript: anti-vinculin antibody shows no band in Western blots of vcl (-/-) mouse embryonic fibroblast cells and only one specific band respectively in vcl (-/-) cells rescued with VV or MV (see supplementary figure 1c).

anti-mouse IgG Alexa Fluor-405 (polyclonal, Invitrogen, A31553; IF 1:500)
application(s): flow cytometry (1-10 µg/ml), immunocytochemistry (1-10 µg/ml), immunofluorescence (1-10 µg/ml), immunohistochemistry (1-10 µg/ml).
species reactivity: mouse

anti-rabbit IgG Alexa Fluor-405 (polyclonal, Invitrogen, A31556; IF 1:500)
application(s): flow cytometry (1-10 µg/ml), immunocytochemistry (1-10 µg/ml), immunofluorescence (1-10 µg/ml), immunohistochemistry (1-10 µg/ml).
species reactivity: rabbit

anti-rabbit IgG Alexa Fluor-488 (polyclonal, Invitrogen, A21441; IF 1:500)
application(s): immunocytochemistry (2 µg/ml), immunofluorescence (2 µg/ml), immunohistochemistry (1-10 µg/ml).
species reactivity: rabbit

anti-rat IgG Alexa Fluor-488 (polyclonal, Invitrogen, A11006; IF 1:500)
application(s): flow cytometry (1-10 µg/ml), immunocytochemistry (4 µg/ml), immunofluorescence (4 µg/ml).
species reactivity: rat

anti-mouse IgG Alexa Fluor-568 (polyclonal, Invitrogen, A11004; IF 1:500)
application(s): flow cytometry (1-10 µg/ml), immunocytochemistry (2 µg/ml), immunofluorescence (2 µg/ml), immunohistochemistry (frozen) (assay dependent concentration).
species reactivity: mouse

anti-rabbit IgG Alexa Fluor-568 (polyclonal, Invitrogen, A11036; IF 1:500)
application(s): immunocytochemistry (4 µg/ml), immunofluorescence (4 µg/ml), immunohistochemistry (1:2000).
species reactivity: rabbit

anti-mouse IgG Alexa Fluor-647 (polyclonal, Invitrogen, A21235; IF 1:500)
application(s): flow cytometry (1-10 µg/ml), immunocytochemistry (2 µg/ml), immunofluorescence (2 µg/ml), immunohistochemistry (assay dependent).
species reactivity: mouse

anti-mouse IgG HRP (polyclonal, BioRad, 170-6516; WB 1:10000)
recommended dilution: 1:3000

anti-rabbit IgG HRP (polyclonal, BioRad, 170-6515; WB 1:10000)
recommended dilution: 1:3000

Eukaryotic cell lines

Policy information about cell lines

Cell line source(s)

HL-1 cells (Merck, SCC065), AmphoPack 293 cell line (Clontech - Takara Bio Europe, 631505).
Talin-deficient mouse kidney fibroblasts ((tln1−/−tln2−/−) were generated by transducing SV40 large T immortalized tln1(f/f) tln2(−/−) cells with Cre recombinase and isolating a clonal cell line (Theodosiou et al, Elife, 2016).
Vinculin-deficient mouse embryonic fibroblasts (vinc(−/−)) were generated by transducing SV40 large T immortalized fibroblast with a loxP flanked vinculin gene (vinc(f/f), Thievessen et al, J Cell Biol, 2013) with Cre recombinase and isolating a clonal cell line with the most similar morphological features to the original vinc(f/f). Vinc(f/f) cells were a gift from Clare Waterman and Ingo Thievessen.

Authentication

Western Blot and immunostainings were performed on vinc(−/−) to confirm V/M deficiency. HL-1 cells were directly ordered from the indicated supplier and characteristic twitching at high seeding densities was observed. Tln1(−/−)tln2(−/−) cells are published (Theodosiou et al, Elife, 2016) and display the described characteristic phenotype of e.g. cell rounding and weak cell-matrix adhesion. The AmphoPack 293 cell line was ordered from the indicated supplier and was not further authenticated.

Mycoplasma contamination

All cell lines were tested negative for mycoplasma by PCR testing.

Commonly misidentified lines
(See ICLAC register)

No commonly misidentified cell line was used.

Animals and other organisms

Policy information about studies involving animals; ARRIVE guidelines recommended for reporting animal research

Laboratory animals

C57BL/6N and Tg(Nes-cre)1Wme mouse strains were used to generate the metavinculin-deficient mouse strain in the course of this study.
Male and female, knock-out (−/−) and control (+/+) mice, aged between six and thirteen months, were used for the phenotype

	analysis. TAC experiment was performed on eight weeks old, knock-out and control male mice.<br>Mice were generated and housed under SPF barrier conditions at the animal facility of the Max Planck Institute of Biochemistry in Martinsried, Germany. Room temperature: 22 ± 1.5°C, relative humidity: 55 ± 5%, lighting: artificial with a light:dark cycle of 14:10 hours.
Wild animals	The study did not involve wild animals.
Field-collected samples	The study did not involve samples collected from the fields.
Ethics oversight	Animals were housed and all experiments were performed in accordance with the study protocol approved by the Government of Upper Bavaria, Germany (No. 55.2-1-54-2532-77-2015).

Note that full information on the approval of the study protocol must also be provided in the manuscript.
